# NAT10 acetylates BCL-XL mRNA to promote the proliferation of multiple myeloma cells through PI3K-AKT pathway

**DOI:** 10.3389/fonc.2022.967811

**Published:** 2022-08-01

**Authors:** Yuanjiao Zhang, Zhendong Deng, Shanliang Sun, Siyuan Xie, Mingmei Jiang, Bing Chen, Chunyan Gu, Ye Yang

**Affiliations:** ^1^ Nanjing Hospital of Chinese Medicine Affiliated to Nanjing University of Chinese Medicine, Nanjing, China; ^2^ School of Medicine & Holistic Integrative Medicine, Nanjing University of Chinese Medicine, Nanjing, China; ^3^ National and Local Collaborative Engineering Center of Chinese Medicinal Resources Industrialization and Formulae Innovative Medicine, Nanjing University of Chinese Medicine, Nanjing, China; ^4^ Department of Hematology, The Affiliated Drum Tower Hospital of Nanjing University of Chinese Medicine, Nanjing, China

**Keywords:** multiple myeloma, NAT10, acetylation, BCL-XL, PI3K-AKT

## Abstract

Multiple myeloma (MM) is a clinically distinctive plasma cell malignancy in the bone marrow (BM), in which epigenetic abnormalities are featured prominently. Epigenetic modifications including acetylation have been deemed to contribute to tumorigenesis. N-acetyltransferase 10 (NAT10) is an important regulator of mRNA acetylation in many cancers, however its function in MM is poorly studied. We first analyzed MM clinical databases and found that elevated NAT10 expression conferred a poor prognosis in MM patients. Furthermore, overexpression of NAT10 promoted MM cell proliferation. The correlation analysis of acRIP-seq screened BCL-XL (BCL2L1) as a significant downstream target of NAT10. Further RNA decay assay showed that increased NAT10 improved the stability of BCL-XL mRNA and promoted protein translation to suppress cell apoptosis. NAT10 activated PI3K-AKT pathway and upregulated CDK4/CDK6 to accelerate cellular proliferation. Importantly, inhibition of NAT10 by Remodelin suppressed MM cell growth and induced cell apoptosis. Our findings show the important role of NAT10/BCL-XL axis in promoting MM cell proliferation. Further explorations are needed to fully define the potential of targeting NAT10 therapy in MM treatment.

## Introduction

Multiple myeloma (MM) is a hematological cancer characterized by clonal expansion of plasma cells secreting large amounts of monoclonal immunoglobulins (1). Currently, MM remains incurable and inevitably relapses due to drug resistance, and more importantly, the monoclonal antibodies and CAR-T therapy are limited for MM patients (2). In addition, the rapid disease progression, short survival time and extremely poor prognosis of relapsed and refractory patients are also huge challenges for MM treatment (3). Therefore, there is an urgent need to discover novel therapeutic targets to develop new drugs.

NAT10 belonging to the family of Gcn5-related N-acetyltransferases possesses histone acetyltransferase (HAT) activity (4), which upregulates telomerase activity through transactivation of the human telomerase reverse transcriptase promoter (5). Moreover, NAT10 is an enzyme that catalyzes the acetylation of ac4C on rRNA, tRNA and mRNA (6). Recently, it is reported that NAT10-catalyzed mRNA acetylation in coding sequences can stabilize and increase translation efficiency of mRNA (7). NAT10 is emerging as a critical regulator in the development of various cancers, such as NAT10 increases the stability of mutant p53 to enhance its tumorigenic activity in liver cancer (8) and acetylates p53 at K120 to stabilize p53 by counteracting the effect of Mdm2 to promote cellular proliferation in colorectal cancer (9). The regulation of cell cycle checkpoint through NAT10 acetylation participates in breast cancer development (10). An oncogenic role of NAT10 also has been validated in hematological malignancies, such as acute myeloid leukemia (4). However, the functions of NAT10 in MM are still not well elaborated.

This study aimed to investigate the potential involvement of NAT10 in the pathogenesis of MM. We found that NAT10 directly enhanced mRNA acetylation and promoted BCL-XL protein translation to inhibit cell apoptosis, leading to activation of PI3K-AKT pathway and CDK4/CDK6 to promote MM cell proliferation. Inhibition of NAT10 by Remodelin suppressed MM cell growth. Our work suggests that NAT10 may be a potentially promising therapeutic target for MM treatment.

## Materials and methods

### Gene expression profiling

The gene expression profiling (GEP) of MM patients were obtained from the GEO database as previously described (11, 12). The examination of The Cancer Genome Atlas (TCGA) datasets using gene expression profiling interactive analysis Gene Expression Profiling Interactive Analysis (GEPIA) webserver (http://gepia.cancer-pku.cn/).

### Antibodies and reagents

The primary antibodies used in this study were at the dilutions of 1:1000 as follows: NAT10 (13365-1-AP, Proteintech), ac4C (ab252215, Abcam), AKT (9272s, Cell Signaling Technology), p-AKT (4058s, Cell Signaling Technology), BCL-XL (2762s, Cell Signaling Technology), Bax (2774s, Cell Signaling Technology), PARP (9542S, Cell Signaling Technology), Cleaved Caspase-3 (9661S, Cell Signaling Technology), β-actin (60008-1-Ig, Proteintech), CDK4 (11026-1-AP, Proteintech), CDK6 (14052-1-AP, Proteintech).

The second antibodies included goat anti-Rabbit IgG(H+L) HRP (FMS-Rb01, Fcmacs) or mouse (S0002, Affinity) were at the dilutions of 1:5000.

Remodelin was purchased from CSNpharm (Chicago, USA). Puromycin was obtained from Merck KGaA (Darmstadt, Germany). Trizol reagent (YEASEN, Shanghai), Hifair 1st Strand cDNA Synthesis SuperMix for qPCR (gDNA digester plus) (YEASEN, Shanghai), SYBR Green PCR master mix (YEASEN, Shanghai).

### Cell lines and culture

Human MM cell lines including KMS28-PE and OPM2 were cultured in RPMI-1640 with 10% fetal bovine serum, 100 U/mL penicillin, and 100 µg/mL streptomycin (Biological Industries, Israel) in a 37°C humidified incubator with 5% CO_2._

### Plasmids and transfection

The plasmids containing the human NAT10 cDNA were purchased from TranSheepBio (Shanghai, China). The NAT10 coding sequence was cloned into the lentiviral vector, pTSB carrying Flag tag. MM cells were transfected using lentivirus as described previously (13).

### Electroporation method

BTXpress Cytoporation Media T4 (BTX, 47-0003) was used to deliver siRNA into cells according to the Manufacturer’s manual (14).

Sequences of siRNA were as following: negative control (sense 5’-UUCUCCGAACGUGUCACGUTT-3’ and anti-sense 5’-ACGUGACACGUUCGGAGAATT-3’); NAT10 (sense 5’- GCAUGGACCUCUCUGAAUATT-3’ and anti-sense 5’- UAUUCAGAGAGGUCCAUGCTT-3’).

### Cell proliferation and viability assay

MM cells (1.5 x 10^3^/well) were seeded in a 96-well plate. CCK8 (Beyotime) reagent was added to each well and incubated for 24, 48 and 72 h, respectively. The absorbance was measured at 450 nm using a microplate reader (Varioskan LUX,Thermo).

### Colony formation

MM cells (1 x 10^4^/well) were seeded in a 24-well plate with 0.5 mL of 0.33% agar/RPMI 1640 supplemented with 10% FBS. The medium was added twice per week for 2 weeks. Colonies were imaged and counted.

### Flow cytometry analysis of cell cycle and apoptosis

Cell cycle and apoptosis were performed according to previous report (15) and analyzed by flow cytometry (Merck Millipore, Germany).

### ac4C detection by dot blot

Dot blot was conducted referring to the method described previously (14). Anti-ac4C antibody was used for dot blot.

### Quantitative PCR

Quantitative PCR (qPCR) was performed as previously described (13).

Sequences of primers were as following: GAPDH: GGGGAGCCAAAAGGGTCATCATC, GACGCCTGCTTCACCACCTTCTTG; BCL-XL: GCCACTTACCTGAATGACCACC, AACCAGCGGTTGAAGCGTTCCT.

### RNA decay assay

MM cells were treated with mRNA transcription inhibitor Actinomycin D (5 μg/mL) (MCE, HY-17559) for 0, 2, 4, 6 h. BCL-XL mRNA was analyzed by qPCR method.

### Statistical analysis

Data were presented as the mean ± standard deviation and analyzed by using a two-tailed Student’s t-test (2 groups) and one-way ANOVA for multiple comparisons. A Kaplan–Meier curve and Log-rank test were employed to determine MM patient survival. *p*<0.05 (*), *p*<0.01 (**) and *p*<0.001 (***) were considered to indicate statistically significant differences.

## Results

### Elevated NAT10 is associated with poor survival in MM patients and promotes MM cell proliferation *in vitro*

**Figure 1:**
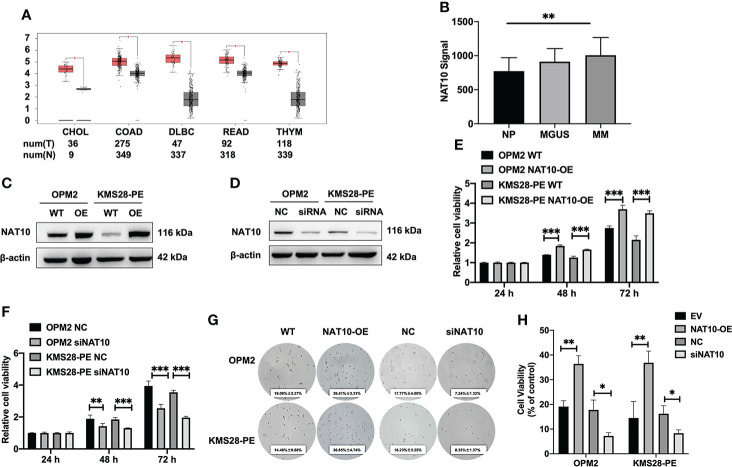
Elevated NAT10 is relevant to poor survival of MM patients and promotes MM cell proliferation. **(A)** Boxplots of high-expressing NAT10 in tumors (T) from CHOL, COAD, DLBC, READ, THYM and normal controls (N). **(B)** NAT10 mRNA levels were significantly elevated in MM patients of GSE6477 dataset. The signal level of NAT10 was shown on the y-axis. The groups of healthy donors with normal BM NP (n=15), MGUS (n=22) and MM (n=69) were displayed on the x-axis respectively. **(C)** Confirmation of NAT10 expression in NAT10-OE MM cells by WB test. **(D)** Confirmation of NAT10 expression in siNAT10 MM cells by WB test. **(E)** CCK8 assay showed increased NAT10 enhancing cellular proliferation**. (F)** CCK8 assay showed siNAT10 impeding cell growth in MM cells. **(G)** Images of representative soft agar plates indicated accelerated clonogenic growth of NAT10-OE cells and suppressed clonogenic growth of siNAT10 cells compared to NC cells. **(H)** Statistical analysis of the long-term proliferation ability of NAT10-OE or siNAT10 MM cells for clonogenic soft agar assay. The data are expressed as mean ± SD. *p < 0.05, **p < 0.01, ***p < 0.001. CHOL, Cholangio carcinoma; COAD, Colon adenocarcinoma; DLBC, Lymphoid Neoplasm Diffuse Large B-cell Lymphoma; READ, Rectum adenocarcinoma; THYM, Thymoma.

The examination of TCGA datasets using GEPIA webserver shows that NAT10 is highly expressed in a variety of cancers (*p*<0.05) (**Figure 1A**). We also interrogated the GEP of NAT10 dataset from normal plasma (NP) cells, monoclonal gammopathy of undetermined significance (MGUS) and MM bone marrow plasma cells, showing that NAT10 expression in patients of MGUS (n=22) and MM (n=69) was obviously increased compared with NP (n=15) (*p*=0.0027; GSE6477) (**Figure 1B**). Then, we constructed stably overexpressing (OE) NAT10 MM cell lines (OPM2 and KMS28-PE) by lentivirus-based method and knocked down NAT10 expression by siRNA technology, which were validated by WB analysis (**Figures 1C, D**). CCK8 assay showed that the cellular proliferation capacity was enhanced in NAT10-OE MM cells relative to WT cells (*p*<0.001) (**Figure 1E**). On the contrary, silencing NAT10 inhibited cell proliferation compared with negative control (NC) cells (*p*<0.001) (**Figure 1F**). Consistently, a clonogenic soft agar assay indicated that interfering the expression of NAT10 significantly altered long-term proliferation of MM cells (*p*=0.0180, *p*=0.1024, *p*=0.0091, *p*=0.0178) (**Figures 1G, H**). These findings support that NAT10 acts as an oncogene stimulating MM cell growth.

### NAT10 acetylates mRNA to regulate the progression of MM

**Figure 2:**
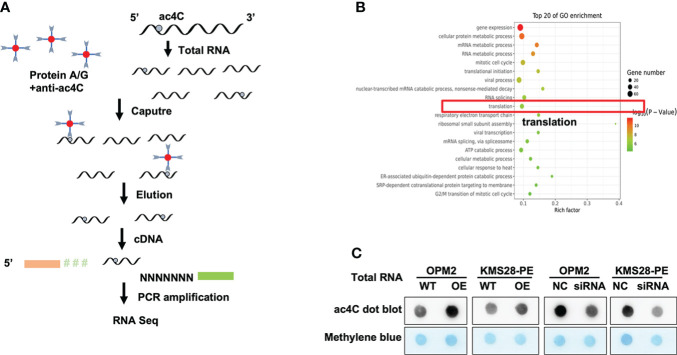
NAT10 acetylates mRNA to regulate the progression of MM. **(A)** Schematic of acRIP-seq. **(B)** GO analysis of acRIP-seq. **(C)** Dot bolt showed that NAT10 overexpression led to higher mRNA acetylation level, and converse result was observed in siNAT10 MM cells.

NAT10 is an acetyltransferase involved in N4-Acetylcytidine (ac4C) modification on tRNA and 18S rRNA (16) and mRNA (17), which may further affect RNA stability and gene expression (7). We used acetylated RNA immunoprecipitation and sequencing (acRIP-seq) to assess the transcriptomic distribution and turnover of ac4C by NAT10 (**Figure 2A**). The acRIP-seq results from KMS28-PE WT and NAT10-OE cells showed that ac4C-enriched mRNA genes were upregulated in NAT10-OE cells compared to WT cells. Gene ontology (GO) analysis of these genes confirmed that gene expression, cellular protein metabolic process, mRNA metabolic process and translation were the top enriched GO terms (**Figure 2B**). Meanwhile, the acetylation level of total RNA in MM cells by dot blot method showed that elevated NAT10 increased ac4C level, while silencing NAT10 resulted in decreased ac4C acetylation in MM cells (**Figure 2C**). Here, it is speculated that NAT10 accelerates the progression of MM by catalyzing mRNA acetylation.

### NAT10 acetylates BCL-XL mRNA to enhance translation efficiency

**Figure 3:**
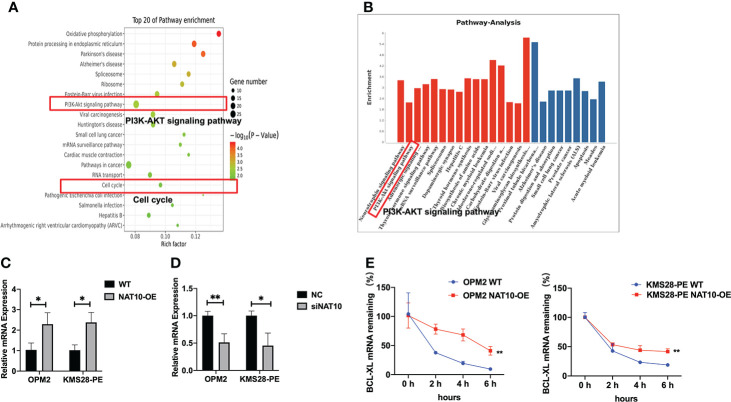
NAT10 acetylates NAT10 mRNA to enhance translation efficiency. **(A)** KEGG pathway enrichment analysis of acRIP-seq. **(B)** Pathway analysis: upregulated pathway in red, downregulated pathway in blue. **(C)** BCL-XL mRNA expression was measured in WT and NAT10-OE MM cells by qPCR. **(D)** BCL-XL mRNA expression was measured in NC and siNAT10 MM cells by qPCR. **(E)** RT-qPCR was performed to detect BCL-XL mRNA stability in OPM2 and KMS28-PE cells with the treatment of Actinomycin D (5 μg/mL). The data are expressed as mean ± SD. *p < 0.05, **p < 0.01, ***p < 0.001.

In order to further screen the downstream targets of NAT10, we also analyzed the Kyoto Encyclopedia of Genes and Genomes (KEGG) pathway results. There were top 20 most significantly enriched pathways, such as the PI3K-AKT signaling and cell cycle pathways (**Figure 3A**). In addition, KEGG pathway analysis showed that the PI3K-AKT pathway was significantly upregulated upon NAT10 overexpression, and BCL-XL was distinguished among the genes associated with the upregulated PI3K-AKT pathway (**Figure 3B**). More importantly, from the sequencing results, we found that the anti-apoptotic BCL-XL was quite different between NAT10-OE cells and WT cells. Additionally, qPCR analysis validated that BCL-XL was significantly increased in NAT10-OE cells (*p*=0.0298, *p*=0.0130) (**Figure 3C**) while decreased in siNAT10 cells (*p*=0.0085, *p*=0.0177) (**Figure 3D**). The RNA decay assays demonstrated a higher stability of BCL-XL transcripts in NAT10-OE cells than that in WT cells (*p*=0.0021 & *p*=0.0011) (**Figure 3E**). Therefore, BCL-XL mRNA may be the direct acetylated target of NAT10 in MM, which further stabilizes and improves translation efficiency of BCL-XL.

### NAT10 enhances BCL-XL mRNA translation and activates PI3K-AKT pathway to promote the proliferation of MM cells

**Figure 4:**
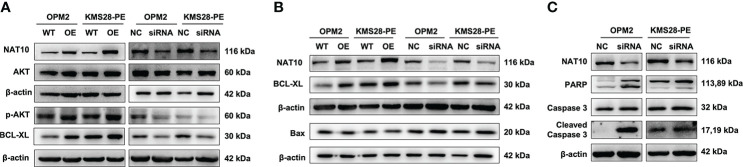
NAT10 acetylates BCL-XL mRNA to enhance its translation efficiency and activates PI3K-AKT pathway to induce anti-apoptosis in MM cells. **(A)** WB examined the expressions of BCL-XL, AKT and p-AKT in OPM2 and KMS28-PE cells. **(B)** WB detected the expressions of BCL-XL and BAX in OPM2 and KMS28-PE cells. **(C)** WB tested the expressions of caspase-3 & cleaved caspase-3 and PARP & cleaved PARP in OPM2 and KMS28-PE cells.

**Figure 5:**
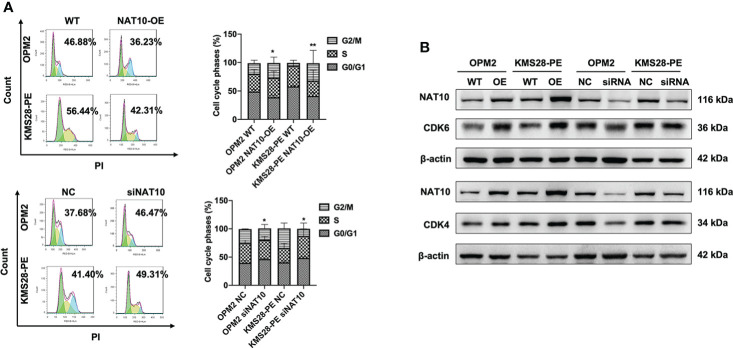
NAT10 regulates cycle distribution and promotes cellular proliferation in MM cells. **(A)** Flow cytometry analysis displayed that NAT10 overexpression decreased G0/G1 phase fraction and siNAT10 increased G0/G1 phase fraction in MM cells. **(B)** WB assays indicted that NAT10 upregulated CDK4 and CDK6 protein expressions and siNAT10 decreased CDK4 and CDK6 protein expressions in MM cells. The data are expressed as mean ± SD. *p < 0.05, **p < 0.01.

To further elucidate the downstream targets and pathways regulated by NAT10, we detected the protein expressions of BCL-XL and upstream pro-survival protein AKT of BCL-XL, whose activity was regulated by phosphorylation. WB results confirmed that BCL-XL was increased in NAT10-OE cells while decreased in siNAT10 cells compared to control cells, respectively. Likewise, the expression of p-AKT not total AKT was increased in NAT10-OE cells while decreased in siNAT10 cells (**Figure 4A**). The increased p-AKT activates the PI3K-AKT pathway and promotes cell proliferation (18–20) by facilitating the synthesis of anti-apoptotic proteins or phosphorylating and inactivating pro-apoptotic Bcl-2-related death-initiating proteins (21). We subsequently verified that the pro-apoptotic protein BAX was decreased upon NAT10 overexpression, and vice versa (**Figure 4B**). Furthermore, the expressions of cleaved-PARP and cleaved-Caspase 3 were increased upon silencing NAT10 (**Figure 4C**). As apoptosis is closely related to cell cycle in cancer cells (22), we next employed flow cytometric analysis to examine cell cycle distribution. A decrease in G0/G1 fraction was observed in NAT10-OE cells (*p*=0.0255, *p*=0.0091) (**Figure 5A**), while an increase in cellular G0/G1 fraction was observed in siNAT10 cells compared to control cells, respectively (*p*=0.0396, *p*=0.0137) (**Figure 5A**). Consistently, the protein expressions of CDK4 and CDK6 were increased in NAT10-OE cells while decreased in siNAT10 cells compared to control cells, respectively (**Figure 5B**). Collectively, NAT10 not only acetylates and promotes the translation of BCL-XL mRNA thus inhibiting cell apoptosis, but also activates the PI3K-AKT pathway to promote cell proliferation possibly *via* regulating the expressions of CDK4 and CDK6.

### Remodelin impedes MM cell growth *in vitro*

**Figure 6:**
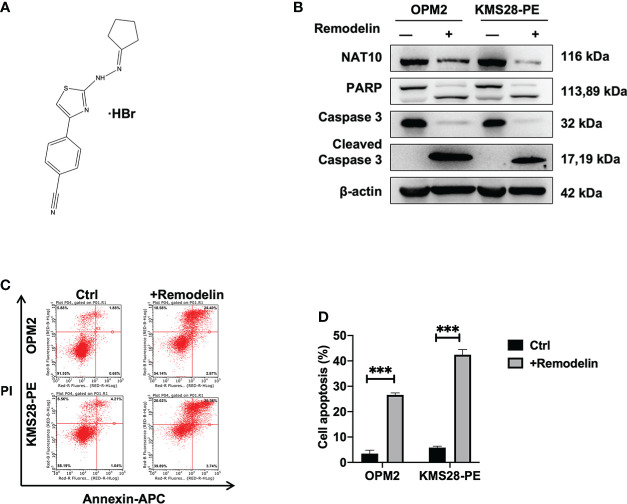
Remodelin impedes MM cell growth *in vitro*. **(A)** The structure of Remodelin. **(B)** WB analysis confirmed that Remodelin increased the expressions of apoptotic proteins: caspase-3 & cleaved caspase-3 and PARP & cleaved PARP. **(C, D)** Flow cytometry analysis indicated that Remodelin facilitated cell apoptosis. The data are expressed as mean ± SD. ***p < 0.001.

We followed to explore the therapeutic potential by targeting NAT10 in MM cells using Remodelin, which is a small molecule inhibitor with the functions of inhibiting NAT10 (**Figure 6A**) and sensitizing tumor cells to chemotherapy (23). WB results showed that Remodelin significantly promoted the expressions of cleaved-PARP and cleaved-Caspase 3 compared to non-treated cells (**Figure 6B**). Furthermore, flow cytometry analysis showed that Remodelin evidently induced apoptosis in MM cells (*p*<0.001) (**Figures 6C, D**). The above data suggest that targeting NAT10 may be a promising strategy for MM treatment.

## Discussion

As a member of the GCN5-related N-acetyltransferase superfamily, NAT10 is involved in the regulation of telomerase activity, DNA damage repair, apoptosis resistance, and cell cycle regulation (24). NAT10 is mostly investigated in solid cancers, but less in hematological cancers. In this study, we found that high expression of NAT10 was associated with poor prognosis in MM patients. Based on the clinical data, we conceived that NAT10 might be a potential target in MM.

NAT10 is the first identified acetylation regulator maintaining efficient translation and stabilizing mRNA by forming ac4C on mRNA (25). More importantly, our ac4C functional analysis revealed an intrinsic role of NAT10 in promoting mRNA stability and translation. The ac4C helps to maintain translation fidelity, and the distal conformation of the N4-acetyl side chain of ac4C is responsible for avoiding misinterpretation of the isoleucine AUA codon during protein translation (26). It also can improve translation efficiency and stability of mRNA. A recent study demonstrates that ac4C on tRNA in plants can improve translation efficiency and fidelity (27), while ac4C plays an important role in maintaining the stability of tRNA that is associated with the high thermotolerance of cells (28). The ac4C is involved in the occurrence of various diseases including cancer (6), inflammation (29), metabolic diseases (30), autoimmune diseases (31). Our study indicated that increased NAT10 led to high ac4C level, while silencing NAT10 decreased ac4C acetylation in MM cells.

PI3K-AKT pathway is a signaling pathway for cell survival involved in multiple cellular processes, especially in cancer development (32). Activation of the downstream transcription factors of PI3K/Akt signaling pathway promotes the synthesis of anti-apoptotic proteins or phosphorylation and inactivation of pro-apoptotic Bcl-2-associated death-initiating proteins. The functional cooperation between PI3K-AKT and Bcl-2 family proteins has become an important mechanism to prevent apoptosis and promote tumorigenesis (33, 34). Apoptosis is a normal physiological process composed of multi-step complex pathways of programmed cell death that is necessary for maintaining cellular homeostasis (35). Two main pathways are related to apoptosis: the extrinsic pathway, which is activated by signals from pro-apoptotic receptors on the cell surface; the intrinsic pathway, which involves disruption of mitochondrial membrane integrity (36). As an anti-apoptotic protein, BCL-XL belongs to the Bcl-2 protein family. The Bcl-2 family controls apoptosis-mediated mitochondrial outer membrane permeability (MOMP) (37), which is a protein playing a vital role in regulating apoptosis (38). Intriguingly, BCL-XL is involved in the pathogenesis of hematological malignancies, for which it maybe a potential biomarker (39).

Dysregulation of cell cycle is a common feature in human cancers, and the cell cycle is closely related to apoptosis (40). There are many similar features between mitosis and apoptosis, indicating a direct link between the cell cycle and apoptosis (41). Our data showed that the G0/G1 phase was decreased and the expressions of CDK4 and CDK6 were increased upon NAT10 overexpression in MM cells. Moreover, we found that anti-apoptotic BCL-XL was increased to inhibit the downstream apoptotic protein BAX, while the upstream p-AKT was elevated to activate PI3K-AKT pathway. These results suggest that maintaining the stability of anti-apoptotic proteins may further induce the activation of PI3K-AKT pathway resulted in MM cell proliferation. Due to the fact that different myeloma cells have different genetic backgrounds (shown as **Supplementary Table 1**), KMS28-PE cell line was less sensitive to genetic manipulation than OPM2 cell line. However, the results in both cell lines were consistent.

Inducing apoptosis is an important way for the treatment of cancer. It is known that Romedelin improves nuclear structure, chromatin organization, and alleviates DNA damage (42). Emerging evidences have shown that Remodelin inhibits cell proliferation, migration and induces cell cycle arrest or apoptosis in various cancer cells (43). We observed that Remodelin suppressed MM cell proliferation and induced apoptosis by inhibiting NAT10 activity *in vitro*, proving the potential of Romedlin for clinical applications. It is worth further exploration to discover specific inhibitors targeting NAT10.

## Conclusion

**Figure 7:**
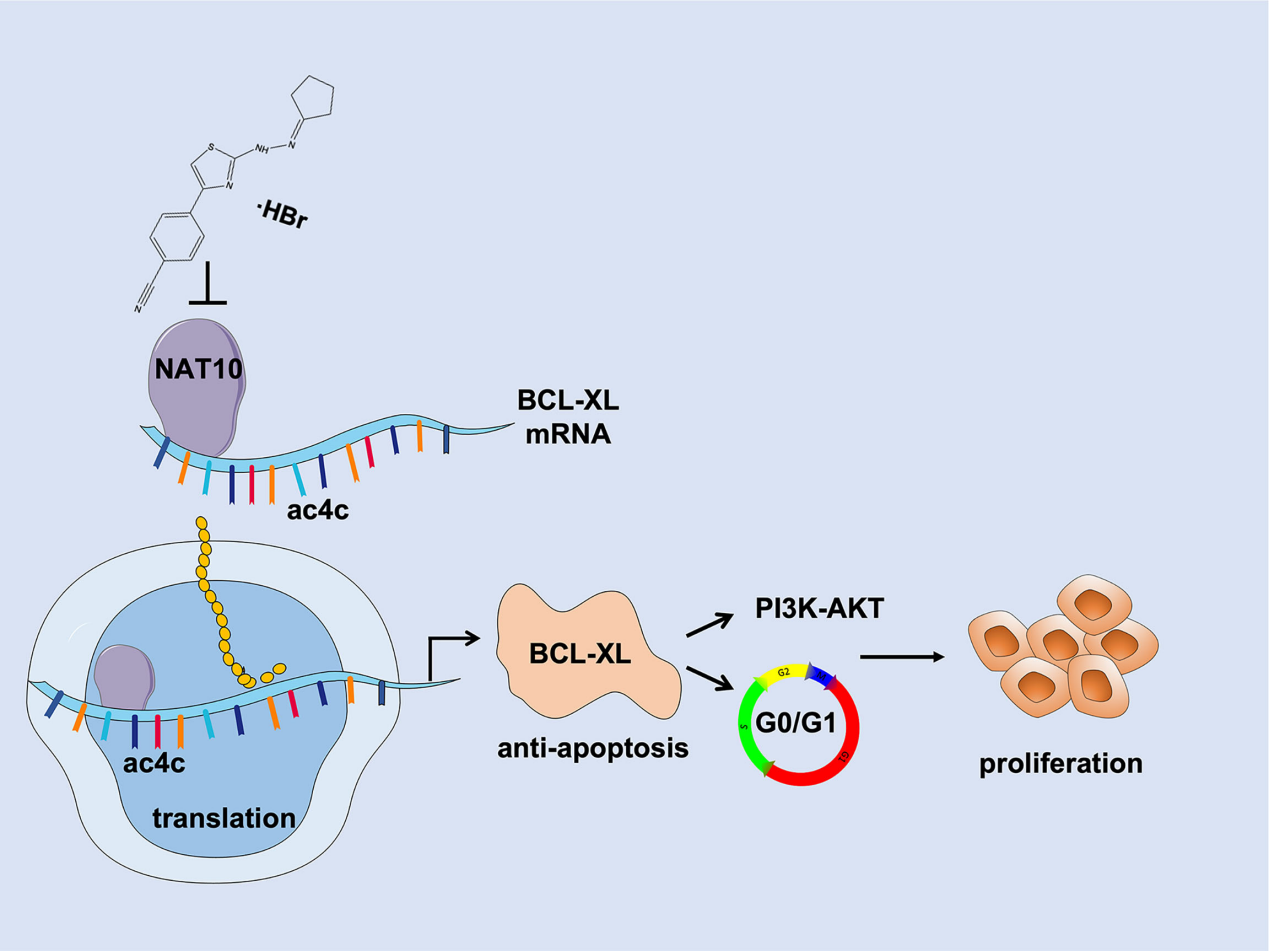
Schematic depiction illustrates that NAT10 is a promising target for improving MM therapy *via* acetylating and stabilizing BCL-XL mRNA to enhance its expression, which inhibits MM cell apoptosis and activates PI3K-AKT pathway thus promoting MM progression and proliferation.

In summary, our findings indicate that NAT10 acetylates and stabilizes BCL-XL mRNA to increase translation efficiency leading to elevated BCL-XL expression, which suppresses MM cell apoptosis and activates PI3K-AKT pathway thus promoting cell cycle progression and proliferation during MM malignancy. Targeting NAT10/BCL-XL axis may be a promising strategy for MM treatment (**Figure 7**).

## Data availability statement

The datasets presented in this study can be found in online repositories. The names of the repository/repositories and accession number(s) can be found in the article/supplementary material.

## Author contributions

YY, CG and BC supervised the project, conceived and edited the manuscript. YZ and CG drafted the manuscript. YZ, ZD and SX performed the experiments. YZ, SS and MJ performed bioinformatics and data analysis. All authors contributed to the article and approved the submitted version.

## Funding

This work was supported by Natural Science Foundation of Jiangsu Province BK20200097 (to CG); National Natural Science Foundation of China 82103985 (to SS); A Project Funded by the Priority Academic Program Development of Jiangsu Higher Education Institutions (Integration of Chinese and Western Medicine); Jiangsu Postgraduate Research and Practice Innovation Program KYCX20_1451 (to YZ) and KYCX22_1984 (to ZD).

## Acknowledgments

The authors acknowledge all the participants who generously gave their help for this study and kind assistance from the experiment center for science and technology of Nanjing University of Chinese Medicine.

## Conflict of interest

The authors declare that the research was conducted in the absence of any commercial or financial relationships that could be construed as a potential conflict of interest.

## Publisher’s note

All claims expressed in this article are solely those of the authors and do not necessarily represent those of their affiliated organizations, or those of the publisher, the editors and the reviewers. Any product that may be evaluated in this article, or claim that may be made by its manufacturer, is not guaranteed or endorsed by the publisher.

## Abbreviations

MM, Multiple myeloma; BM, bone marrow; NAT10, N-acetyltransferase 10; GEP, gene expression profilin; acRIP-seq, acetylated RNA immunoprecipitation sequencing; WB, Western Blot; TCGA, The Cancer Genome Atlas; GEPIA, Gene Expression Profiling Interactive Analysis; siRNA, small interfering RNA, NP, normal plasma; MGUS, monoclonal gammopathy of undetermined significance; GO, Gene ontology; KEGG, Kyoto Encyclopedia of Genes and Genomes; CHOL, Cholangio carcinoma; COAD, Colon adenocarcinoma; DLBC, Lymphoid Neoplasm Diffuse Large B-cell Lymphoma; READ, Rectum adenocarcinoma; THYM, Thymoma.
