# Supplementary material for: NAT10 acetylates BCL-XL mRNA to promote the proliferation of multiple myeloma cells through PI3K-AKT pathway
Source: Front Oncol. 2022 Aug 1;12:967811. doi: 10.3389/fonc.2022.967811 (PMC9376478; doi:10.3389/fonc.2022.967811)
Supplement: Supplementary file 1 [file Table_1.docx]

**Supplementary table 1 Common Genetics of MM cell lines**

| **Cell Line** | **Ig Tx** | **BIRC2/3** | **CDKN2C** | **FGFR3** | **KRAS** | **NRAS** | **TP53** |
| --- | --- | --- | --- | --- | --- | --- | --- |
| OPM2 | t(4:14) |  | HD | K650E-Het | Wt | Wt | R175H-homo (CCLE)(cc) |
| KMS-28PE | t(4;14) + t(8;14) | HD |  | Expressed | G12A - homo (cc) |  | G105R-homo (cc) |
