# Supplementary material for: NAT10 acetylates BCL-XL mRNA to promote the proliferation of multiple myeloma cells through PI3K-AKT pathway
Source: Front Oncol. 2022 Aug 1;12:967811. doi: 10.3389/fonc.2022.967811 (PMC9376478; doi:10.3389/fonc.2022.967811)
Supplement: Supplementary file 2 [file Table_2.docx]

**Supplementary table 2 Statistics of Pathway Enrichment**

| **PathwayID** | **PathwayTerm** | | **DifGene** | **AllDifGene** | **GeneInPathway** | **AllGene** | **P-Value** | **FDR** | **Enrichment** | **(-log10P)** |
| --- | --- | --- | --- | --- | --- | --- | --- | --- | --- | --- |
| PATH:04722 | Neurotrophin signaling pathway | 7 | | 107 | 130 | 6782 | 0.00430819 | 0.44059123 | 3.41294033 | 2.36570472 |
| PATH:04151 | PI3K-Akt signaling pathway | | 12 | 107 | 347 | 6782 | 0.00820105 | 0.44059123 | 2.19192545 | 2.08613068 |
| PATH:04261 | Adrenergic signaling in cardiomyocytes | | 7 | 107 | 149 | 6782 | 0.00896764 | 0.44059123 | 2.97773317 | 2.04732168 |
| PATH:04919 | Thyroid hormone signaling pathway | | 6 | 107 | 119 | 6782 | 0.01103106 | 0.44059123 | 3.19579047 | 1.9573829 |
| PATH:03015 | mRNA surveillance pathway | | 5 | 107 | 91 | 6782 | 0.01416214 | 0.44059123 | 3.48259217 | 1.84887101 |
| PATH:03040 | Spliceosome | | 6 | 107 | 130 | 6782 | 0.01651009 | 0.44059123 | 2.92537743 | 1.78225055 |
| PATH:04728 | Dopaminergic synapse | | 6 | 107 | 131 | 6782 | 0.01708689 | 0.44059123 | 2.9030463 | 1.76733702 |
| PATH:05160 | Hepatitis C | | 6 | 107 | 137 | 6782 | 0.02084214 | 0.44059123 | 2.77590559 | 1.68105769 |
| PATH:04918 | Thyroid hormone synthesis | | 4 | 107 | 72 | 6782 | 0.02655689 | 0.44059123 | 3.52128764 | 1.57582284 |
| PATH:01230 | Biosynthesis of amino acids | | 4 | 107 | 73 | 6782 | 0.02776525 | 0.44059123 | 3.47305083 | 1.55649842 |
| PATH:05220 | Chronic myeloid leukemia | | 4 | 107 | 73 | 6782 | 0.02776525 | 0.44059123 | 3.47305083 | 1.55649842 |
| PATH:04960 | Aldosterone-regulated sodium reabsorption | | 3 | 107 | 42 | 6782 | 0.02808692 | 0.44059123 | 4.52736983 | 1.55149591 |
| PATH:04973 | Carbohydrate digestion and absorption | | 3 | 107 | 45 | 6782 | 0.03356564 | 0.44059123 | 4.22554517 | 1.4741051 |
| PATH:05169 | Epstein-Barr virus infection | | 7 | 107 | 201 | 6782 | 0.0390914 | 0.44059123 | 2.20737434 | 1.40791877 |
| PATH:05203 | Viral carcinogenesis | | 7 | 107 | 207 | 6782 | 0.04466304 | 0.44059123 | 2.14339248 | 1.35005169 |
| PATH:00532 | Glycosaminoglycan biosynthesis - chondroitin sulfate / dermatan sulfate | | 2 | 107 | 22 | 6782 | 0.04640358 | 0.44059123 | 5.76210705 | 1.33344856 |
